# Restoring Nature's Backbone

**DOI:** 10.1371/journal.pbio.0040202

**Published:** 2006-06-13

**Authors:** Henry Nicholls

## Abstract

Is it feasible to introduce contemporary species to fulfill the ecological role of species that went extinct thousands of years ago (rewilding) and can it work as an effective conservation strategy?

A herd of bison has just made an extraordinary migration. The distance these animals travelled was huge—nearly 5,000 miles—and their means of transport was highly unorthodox: they flew. The cargo plane took some thirty of these hulking mammals from Elk Island National Park of Canada across Alaskan airspace, over the Bering Strait, and into the Republic of Yakutia. Their ultimate destination: the Lenskiye Stolby Nature Park, a 78,500-hectare reserve in northeast Siberia more commonly known as “Pleistocene Park.”

This is the latest phase of an experiment to explore the impact of large herbivores that once roamed these lands on the biodiversity and integrity of the Siberian steppe ecosystem. One key consequence of putting such creatures back is that they disrupt the snow cover during the winter, exposing the ground to the cold and preserving the permafrost. Without these herbivores, the snow insulates the earth and the permafrost melts, says Sergei Zimov, director of the Northeast Science Station in Cherskii, and the brains behind Pleistocene Park. This could allow microbes to break down vast reserves of carbon contained in the earth, thereby contributing to global warming, he says [ 1]. The return of once-native flora and fauna—so-called “rewilding”—should prevent this and bring the soil much-needed fertilization. “Rewilding will increase the bioproductivity and biodiversity of the landscape,” he predicts.

“If we lose these large predators from the ecosystem, biodiversity is the ultimate loser.”

## On the Offensive

This is all part of a proactive approach to nature being articulated with increasing regularity. Rather than trying to simply ring-fence what wildlife remains, conservationists need to be restoring whole ecologies to something of their former glory, says Josh Donlan, an ecologist at Cornell University (Ithaca, New York, United States). Last year, he and a long list of high-profile conservation biologists penned a controversial commentary in *Nature* in which they laid out the case for rewilding North America—seeding the continent with suitable stand-ins for species that went extinct thousands of years ago [ 2].

Donlan's world would see carefully chosen slivers of North America grazed by giant tortoises, horses, and camels; the stamping ground of elephants in place of five species of mammoth; and African lions in lieu of the extinct American lion that once stalked the continent.

The benefits, they argued, are obvious. It would restore ecological processes that have gone by the wayside, mend broken evolutionary relationships, create a back-up population of some of the planet's most endangered species, and raise huge awareness for the conservation cause. “The obstacles are substantial and the risks are not trivial, but we can no longer accept a hands-off approach to wilderness preservation,” they wrote of their optimistic vision.

There are several compelling illustrations of the importance of big creatures for the integrity of an ecosystem. “There's more and more evidence that large vertebrates are disproportionately important not only for maintaining biodiversity but also for generating biodiversity,” Donlan says ( Box 1). It's examples like these that persuade him of the importance of restoring populations of large vertebrates. “Over the past 30 to 40 years, increasing evidence is showing that if we lose these large predators from the ecosystem, biodiversity is the ultimate loser,” he says.

## Broken Links

The argument for rewilding is also about patching up broken evolutionary links between species. In New Zealand, for example, there are more than 50 endemic “divaricate” plants—species with thin, interwoven branches that form a tangled canopy. One explanation for this unusual structure is that it is an evolutionary adaptation to fend off the herbivorous approaches of the dozen or so species of flightless moa that went extinct with the arrival of humans in New Zealand about 1,000 years ago. Researchers have tested this hypothesis by observing the impact of emus and ostriches—surviving analogues of the extinct moa—on divaricate species where juvenile stems are tangled but adults are not ( Figure 3). The birds removed 30%–70% less foliage from juvenile shoots than adult shoots [ 5]. “A large section of the New Zealand woody flora is specifically adapted to ratite browsing,” says Bill Lee, a plant ecologist at Landcare Research in Dunedin, New Zealand. “We plan to use emu and ostriches in experiments in native ecosystems to examine how they modify ecosystem processes and to investigate their impact on native and introduced plants with different architectures,” he says.

The story is similar in the Mascarenes in the Indian cean, where Aldabran tortoises are being introduced onto a 28-hectare island nature reserve as proxies for the extinct Geochelone inepta and G. triserrata. Several native plant species appear to have evolved distinct juvenile morphological features as a defence against tortoise herbivory. The introduced tortoises are clearly avoiding these species when they are in the juvenile stage, says Vikash Tatayah, fauna manager of the Mauritian Wildlife Foundation (Vacoas, Mauritius).

There are countless other examples of severed links between species. Donlan and his colleagues cite the pronghorn, a deer-like mammal that spent more than four million years on North American grasslands trying to keep one hoof ahead of the now-extinct American cheetah. This key predator almost certainly shaped the pronghorn's astonishing speed, they wrote.

## Benchmarks and Proxies

This sort of proactive vision for conservation raises some tricky questions, notably those of restoration benchmarks. In North America, conservation biologists routinely turn to the arrival of Christopher Columbus in 1492, Donlan says. “This is the default benchmark just because it is.” But, he and his colleagues argued, it would make more ecological sense to think about the arrival of humans on the continent some 13,000 years ago. This is the point at which the human-driven extinction of many large vertebrates contributed to a radical change in the continent's wildlife and a rapid loss of biodiversity, he says.

Elsewhere the appropriate benchmark may be different. David Steadman, curator of ornithology at the Florida Museum of Natural History (Gainesville, Florida, United States), has excavated on dozens of islands across the Pacific Ocean. Soon after the arrival of humans between 30,000 and 1,000 years ago depending on the island, whole swathes of endemic fauna vanish from the fossil record, Steadman says. As many as 2,000 species of bird that would probably exist today quickly wound up on the extinction scrapheap, he says [ 6].

“Large vertebrates are disproportionately important not only for maintaining biodiversity but also for generating biodiversity.”

In such places, the relatively recent arrival of humans with such dramatic effects makes a good case for setting a restoration benchmark. In places like Europe, where humans have been modifying the landscape for far longer, things are not going to be as clear-cut.

Even if there is agreement, there is still a debate to be had over the choice of species for restoration. When a species has disappeared completely, the idea is to use an ecological analogue or “proxy” for the extinct species. In some situations, so little choice remains that the decision is all but made. For example, if scientists ever attempt to restore flightless rail to the Pacific islands that the fossil record suggests had them, they will have only a handful of candidate species. “While it would be nice to be biogeographical purists, we don't have that luxury anymore,” Steadman says. But other settings could have many candidate proxies. It is still not clear whether the candidates should be chosen for their genetic, behavioural, or ecological similarity to the extinct species.

## Virtual Extinctions

For those studying food webs—descriptions of who eats whom—it is the ecological services that a species performs that is crucial. Computer modelling of food webs is a good way to explore the impact of extinctions on an ecosystem ( Figure 4). “You can take a species on the computer and kill it, but you can't in the wild as it's probably illegal,” says Jane Memmott, a community ecologist at the University of Bristol (Bristol, United Kingdom). “We should be conserving ecosystem services and interactions between species,” she says. “It's harder to come up with a food-web recovery plan, but we're definitely moving in that direction.”

One of the more robust findings of such virtual worlds is that removing the most highly connected species causes more secondary, knock-on extinctions than does the removal of species at random. In certain webs, large vertebrates can be highly connected. “We can say they played very important roles and losing them played huge knock-on effects,” confirms Neo Martinez, director of the Pacific Ecoinformatics and Computational Ecology Lab (PEaCE; Berkeley, California, United States). But restoring one or two absent vertebrates to a habitat may do little to repair an altered food web. “The sort of conditions that allowed the vertebrates to play their role just aren't here anymore,” Martinez says. “We just don't have enough knowledge about these systems to predict what would happen. The whole history of trophically oriented biological control is not pretty.”

“The more extravagant rewilding suggestions presuppose that we know what we're doing.”

One way to improve on ecology's predictive power might be to construct “paleo food webs,” collating information from the fossil record to understand the prehistoric interactions between species. Martinez is one of a handful of scientists interested in this approach. There is a wealth of paleobiological evidence that can help resurrect extinct food webs, he says.

Famous fossil ecosystems such as the iconic Burgess Shale are an obvious place to start. “Even by contemporary standards of food webs, these are really good data,” Martinez says. Although much of the bizarre Burgess Shale fauna came to an evolutionary dead end during the Cambrian period, studying the food web of an entire suite of species from a long-gone era could help us to understand how ecosystems have functioned through deep time and reveal general processes that can and cannot be counted on, he notes. “It starts to put envelopes around the plausible dynamics of a system.” If a rewilding initiative were to push that envelope too far, it would be likely to fail. “The more extravagant rewilding suggestions presuppose that we know what we're doing. We don't,” Martinez says. “Not yet.”

## No Alternative

Donlan is well aware that there are substantial biological, social, and economic hurdles to clear if rewilding is to take off. It will take time, careful planning, well-designed experiments, and three stages: first, the restoration of populations of herbivores as is already occurring in the Siberian Pleistocene Park; second, rewilding large protected areas with predators; third, the formation of one or more “ecological history parks” on, for example, vast tracts of North America's Great Plains. The costs and benefits of such proactive conservation must be carefully calculated on a case-by-case basis. “If the costs outweigh the benefits, you don't proceed,” Donlan says. But the conservation community needs to think carefully about these ideas, he says. “There are substantial risks of not doing anything.”

**Box 1. The Loss of Tooth and Claw**

**Figure 1:**
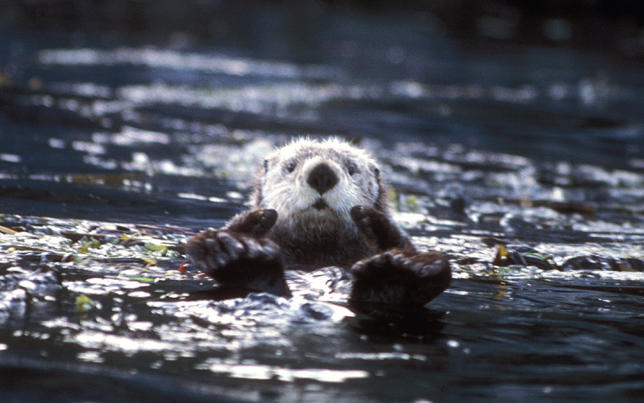
Sea Otters Sea otters are keystone predators in the kelp forest ecosystem of Alaska, keeping invertebrate populations in check and thereby maintaining biodiversity (Photograph: David Menke, US Fish and Wildlife Service)

During the 18th and 19th centuries, overhunting decimated the population of sea otters feeding off the coast of Alaska ( Figure 1). The disappearance of this predator set in motion a top-down cascade that rippled its way through the kelp forest community. Without otters, prey species—marine invertebrates like sea urchins, clams, snails, and crabs—took over, virtually destroying the kelp forests and wiping out countless ecological niches [ 3].

**Figure 2:**
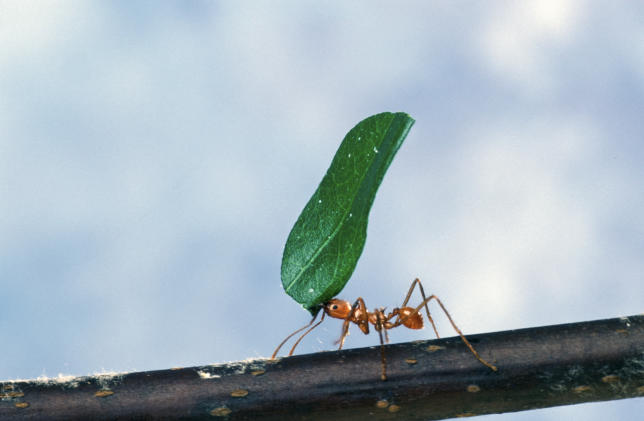
Leaf-Cutter Ants Leaf-cutter ants can take over when predator pressure is removed (Photograph: Scott Bauer, US Department of Agriculture)

Lessons could also be learned from a long-term study in Venezuela. In the 1980s, a valley was flooded as part of a hydroelectric scheme. This created Lake Guri, a 4,300-square-kilometer body of water dotted with hundreds of forested islands of various sizes. Large vertebrates, often predators, struggled to survive on small islands less than 2 hectares in size, and in their absence herbivores like leaf-cutter ants thrived ( Figure 2). Conversely, islands of over 75 hectares could accommodate predators, keeping herbivory in check. By 1997, the density of saplings on small islands was only 37% of that on large islands. Over the next five years, small islands lost 46% of their trees and shrubs, compared with only 32% on large islands [ 4].

**Figure 3:**
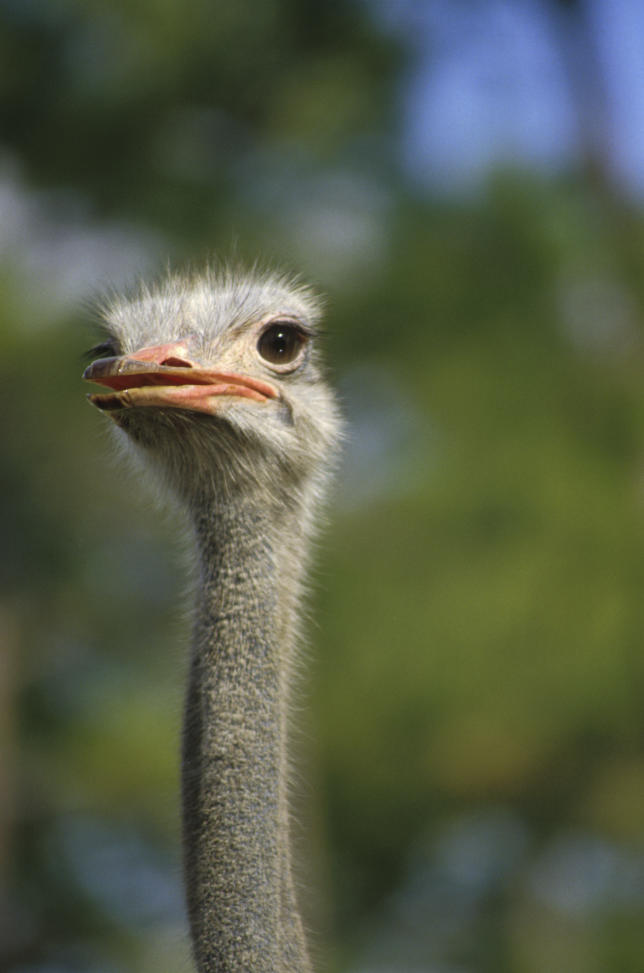
Ostrich The ostrich could fill a similar evolutionary niche to the extinct moas of New Zealand (Photograph: Beth Jackson, US Fish and Wildlife Service)

**Figure 4:**
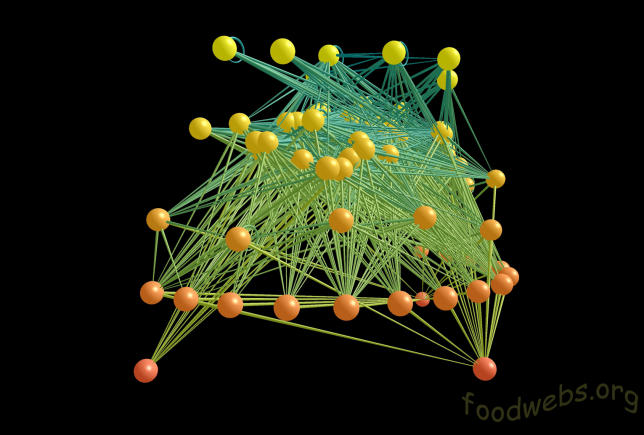
Food Web from a Caribbean Reef (Image created by software written by R. J. Williams and provided by the PEaCE Lab ( http://www.foodwebs.org))

## References

[pbio-0040202-b1] Zimov SA (2005). Pleistocene Park: Return of the mammoth's ecosystem. Science.

[pbio-0040202-b2] Donlan J, Greene HW, Berger J, Bock CE, Bock JH (2005). Re-wilding North America. Nature.

[pbio-0040202-b3] Estes JA, Danner EM, Doak DF, Konar B, Springer AM (2004). Complex trophic interactions in kelp forest ecosystems. Bull Mar Sci.

[pbio-0040202-b4] Terborgh J, Feeley K, Silman M, Nuñez P, Balukjian B (2006). Vegetation dynamics of predator-free land-bridge islands. J Ecol.

[pbio-0040202-b5] Bond WJ, Lee WG, Craine JM (2004). Plant structural defences against browsing birds: A legacy of New Zealand's extinct moas. Oikos.

[pbio-0040202-b6] Steadman DW, Martin PS (2003). The late Quaternary extinction and future resurrection of birds on Pacific Islands. Earth-Science Reviews.

